# The contribution of DNA ploidy to radiation sensitivity in human tumour cell lines

**DOI:** 10.1038/sj.bjc.6690119

**Published:** 1999-02

**Authors:** J L Schwartz, J Murnane, R R Weichselbaum

**Affiliations:** Department of Radiation Oncology, Box 356069, University of Washington, Seattle, WA, 98195, USA; Department of Radiation Oncology, University of California, San Francisco, CA, 94143, USA; Department of Radiation and Cellular Oncology, The University of Chicago, Chicago, 5841 S. Maryland Ave, Chicago, IL, 60637, USA

**Keywords:** predictive assay, DNA ploidy, radiotherapy, chromosome aberrations

## Abstract

The contribution of DNA ploidy to radiation sensitivity was investigated in a group of eight human tumour cell lines. As previous studies suggest, while more aneuploid tumours tend to be more radioresistant, there is no significant relationship between ploidy and radiation sensitivity (SF_2_). The failure to observe a significant effect of ploidy on radiation sensitivity is due to the complex and multifactorial basis of radiation sensitivity. When we determined the relationship between survival and radiation-induced chromosome aberration frequency, a measure independent of most other modifiers of sensitivity, we observed a direct relationship between ploidy and mean lethal aberration frequency. The mean lethal frequency of aberrations increased from about 1 for diploid cells to about 2 for tetraploid cells. The mean lethal frequency of aberrations was independent of DNA repair variations. These observations demonstrate that changes in DNA ploidy are an important contributor to radiation sensitivity variations in human tumour cell lines. Therefore, any battery of predictive assays should include DNA ploidy measurements. © 1999 Cancer Research Campaign


*British Journal of Cancer* (1999) **79**(5/6), 744–747
© 1999 Cancer Research Campaign
Article no. bjoc.1998.0119

### The contribution of DNA ploidy to radiation sensitivity in human tumour cell lines

**JL Schwartz[1], J Murnane[2] and RR Weichselbaum[3]**

[1]Department of Radiation Oncology, Box 356069, University of Washington, Seattle, WA 98195, USA; [2]Department of Radiation Oncology, University of California, San Francisco, CA 94143, USA; [3]Department of Radiation and Cellular Oncology, The University of Chicago, 5841 S. Maryland Ave, Chicago, IL 60637, USA

**Summary** The contribution of DNA ploidy to radiation sensitivity was investigated in a group of eight human tumour cell lines. As previous studies suggest, while more aneuploid tumours tend to be more radioresistant, there is no significant relationship between ploidy and radiation sensitivity ($SF_2$). The failure to observe a significant effect of ploidy on radiation sensitivity is due to the complex and multifactorial basis of radiation sensitivity. When we determined the relationship between survival and radiation-induced chromosome aberration frequency, a measure independent of most other modifiers of sensitivity, we observed a direct relationship between ploidy and mean lethal aberration frequency. The mean lethal frequency of aberrations increased from about 1 for diploid cells to about 2 for tetraploid cells. The mean lethal frequency of aberrations was independent of DNA repair variations. These observations demonstrate that changes in DNA ploidy are an important contributor to radiation sensitivity variations in human tumour cell lines. Therefore, any battery of predictive assays should include DNA ploidy measurements.

**Keywords:** predictive assay; DNA ploidy; radiotherapy; chromosome aberrations

Interindividual variations in tumour response to radiation are thought to reflect in part the intrinsic cellular radiosensitivity of the cells in the tumour (West, 1994). Thus, there has been a lot of interest in trying to understand the basis for variations in inherent radiation sensitivity. There are many different variables that can influence radiation sensitivity and might account for inter-individual differences in radiation sensitivity (Szumiel, 1981; West, 1994). One that has been extensively examined is DNA ploidy. A number of studies have reported that diploid tumours show better responses to radiation therapy than aneuploid cells (reviewed in West, 1994). Other studies, however, find no relationship between ploidy and response. Similarly, in vitro studies on the relationship between radiation sensitivity and chromosome content (reviewed in Szumiel, 1981; Cornforth and Bedford, 1987) have failed to show a consistent relationship between chromosome content and radiosensitivity. One complicating factor is that radiation sensitivity is likely defined by more than one variable, and therefore the influence of ploidy or chromosome content may be masked by differences in other factors such as alterations in DNA repair. The goal of the present study was to investigate the role of ploidy in human tumour cell radiation sensitivity independent of other modifiers of response.

The primary lethal lesion induced by ionizing radiation is a chromosome aberration (Dewey et al, 1971; Carrano, 1973; Bedford et al, 1978; Grote et al, 1981; Cornforth and Bedford, 1987; Bedford, 1991). Presumably, acentric fragments that form as a result of radiation exposure are eliminated from cells due to their inability to properly segregate at anaphase. The resulting loss of genetic information is lethal. This has been shown by many different investigators using a variety of experimental approaches including the direct observation of irradiated cells (Grote et al, 1981). Analysis of chromosome aberrations allows one to examine questions concerning the role of DNA ploidy and chromosome content on radiation sensitivity independent of other factors. For example, variations in sensitivity to DNA damage induction or alterations in DNA repair should influence the number of chromosome aberrations induced, not the relationship between aberration frequency and survival. If DNA ploidy influences radiation sensitivity, then the relationship between aberration frequency and survival will change as ploidy changes. In normal diploid fibroblasts, a single aberration is considered lethal (Cornforth and Bedford, 1987). In hyperdiploid tumour cells, perhaps higher levels of chromosome aberrations may be required to kill the cell. In the present work, we examined the relationship between chromosome aberration frequency and survival in a group of human tumour cell lines. Our analysis confirms the observations that chromosome aberrations are the primary lethal lesion in tumour cells and demonstrates that ploidy influences radiation sensitivity in human tumour cell lines.

### MATERIALS AND METHODS

Eight human tumour cell lines, established from tumour biopsy specimens, were studied (Table 1). One, A549, was derived from a lung adenocarcinoma. The other seven were derived from head-and-neck squamous cell carcinomas (SCC). The methods of establishment, characterizations and growth conditions used for each of the cell lines have been previously described (Weichselbaum et al, 1989; Schwartz, 1992; Russell et al, 1995). All of the cell lines were non-clonal populations that had been maintained in culture

*Received 22 April 1998*
*Revised 4 August 1998*
*Accepted 5 August 1998*

*Correspondence to:* JL Schwartz

**Table 1** Characteristics of cell lines[a]

| Cell line | $SF_2$ | Chromosomes/cell[b] |
|---|---|---|
| SCC-12B.2 | 0.64 | 73 ± 2 (66–76) |
| A549 | 0.82 | 65 ± 3 (55–70) |
| SCC-61 | 0.27 | 47 ± 1 (38–86) |
| SQ-38 | 0.44 | 90 ± 2 (84–99) |
| SQ-20B | 0.51 | 62 ± 1 (43–112) |
| SQ-9G | 0.35 | 57 ± 2 (48–116) |
| SCC-25 | 0.35 | 68 ± 1 (58–94) |
| JSQ-3 | 0.67 | 89 ± 11 (62–122) |

[a]The data for the squamous cell carcinomas are from Weichselbaum et al (1989) and Schwartz (1992). [b]Mean number ± SEM. Range is in parentheses.

for many years. Their radiation sensitivity characteristics are stable, and no large changes in ploidy have been noted. Radiation sensitivity was determined by clonogenic cell survival assay as previously described (Weichselbaum et al, 1989; Russell et al, 1995). Briefly, exponentially growing cultures were trypsinized from stock cultures and between 500 and 40 000 cells were plated in 60-mm tissue culture dishes, allowed to enter exponential growth, and then exposed to either 250 kV X-rays or $^{137}Cs$ γ-rays. Cells were incubated at 37°C for up to 3 weeks, after which the cells were fixed and stained with crystal violet. Only colonies of more than 50 cells were scored as survivors. Survival following a 2 Gy exposure is presented. The survival data from the SCC cell lines has been previously reported (Weichselbaum et al, 1989).

For the cytogenetic analyses, cultures were exposed to graded doses up to 8 Gy and then incubated for 18–30 h, depending on the growth rate of the cell line, to maximize the scoring of cells that were irradiated in $G_1$ (Schwartz, 1992). Two or three different doses were evaluated for each cell line. Cells were arrested in metaphase with a 2-h treatment with 0.2 μM colcemid. Cells were harvested, incubated in 0.075 M potassium chloride for 20 min, and fixed in 3:1 methanol:acetic acid. Fixed cell suspensions were dropped onto slides, air-dried, and stained in a 5% Giemsa solution for later cytogenetic analysis. Cells were analysed for chromosome-type aberrations as described by Savage (1975). Twenty-five to 50 cells per dose-point were analysed. Results are the mean ± SEM of 2–4 experiments. Mean chromosome number was determined from unexposed cultures. The chromosome aberration data for the SCC cell lines was originally published in Schwartz (1992). Survival and chromosome aberration frequency were compared by linear regression analysis.

### RESULTS

The radiation sensitivities of the cell lines are shown in Table 1. Survival following a 2 Gy exposure ($SF_2$) ranged from 0.27 for SCC-61 cells to 0.82 for A549 cells. There is no evidence for any radiation-induced apoptosis in these cells (unpublished observations). By morphologic and flow cytometric analysis, apoptosis levels are less than 10% in the control and in the irradiated cultures. These cell lines apparently die a mitotic death.

Chromosome aberration frequency was compared to survival measured after the same exposure level (Figure 1A). There was a highly significant linear relationship between survival and chromosome aberration frequency ($r^2 = 0.80$, $P = 4.42 \times 10^{-7}$). The slope of the regression was determined for each cell line. From the slope, a measure of the mean lethal aberration frequency for the cell line, that is, the number of aberrations that yields an average of 1 lethal hit per cell (Hall, 1994) was calculated. For diploid fibroblasts, the mean lethal aberration frequency is 1 aberration/cell (Cornforth and Bedford, 1987). The mean lethal aberration frequency varied from 1.2 aberrations/cell for SCC-61 cells to 2.6 aberrations/cell for JSQ-3 cells. When the data for all the cell lines was combined, the mean lethal aberration frequency was 1.7 ± 0.2 aberrations/cell. The extrapolation number for these cells was 1.1 ± 0.1.

We initially used chromosome number as a measure of relative DNA content. Chromosome number was determined for each cell line using the same slides used for the aberration analysis. The

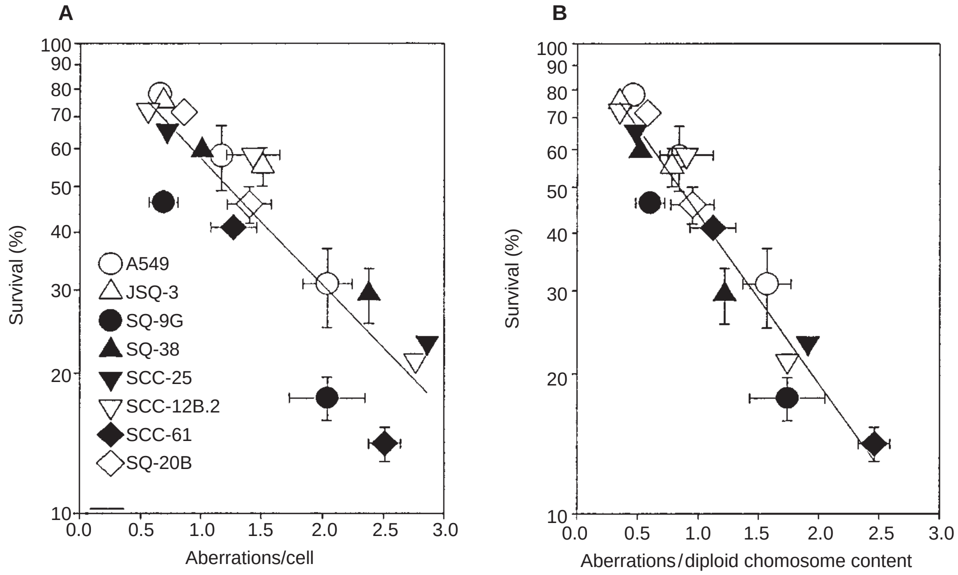

**Figure 1** The relationship between chromosome aberration frequency and survival in human tumour cell lines. (**A**) Uncorrected aberration data. (**B**) Aberration frequencies corrected for chromosome number and expressed as aberrations/diploid chromosome content. Each symbol is a different tumour cell line. Mean ± SEM and linear regression line is shown

© Cancer Research Campaign 1999 

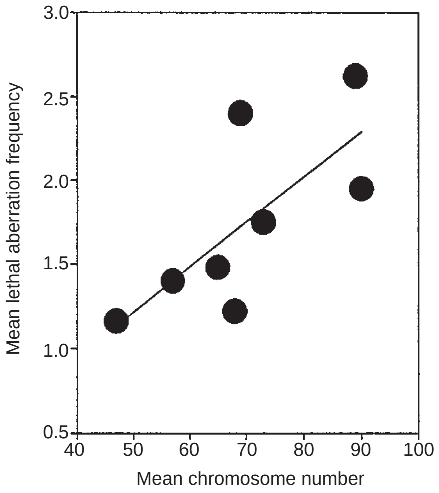

**Figure 2** The relationship between mean chromosome number and mean lethal aberration frequency in human tumour cell lines.

non-clonal cell lines we studied have been maintained almost continually for many years. DNA ploidy levels were fairly stable. That is evident from the relatively small standard errors around each mean chromosome number (Table 1). Thus, the potential complicating effects of a large subset of cells with different chromosome constitutions were not a problem for our study. Mean chromosome number varied from 47 for SCC-61 cells to 90 for SQ-38 (Table 1). The average for all eight cell lines is 69.8 ± 4.8 chromosomes/cell. Mean chromosome number was compared to both plating efficiency and cell doubling time, data previously reported in Schwartz (1992). There was no significant relationship between mean chromosome number and plating efficiency ($r^2$ = 0.01, $P$ = 0.80). There was, however, a significant inverse relationship between mean chromosome number and doubling time ($r^2$ = 0.50, $P$ = 0.046). Paradoxically, cells with greater numbers of chromosomes cycled more rapidly.

There was no significant relationship between mean chromosome number and $SF_2$ ($r^2$ = 0.18, $P$ = 0.30). As shown in Figure 2, however, there was a significant relationship between mean chromosome number and the mean lethal aberration frequency calculated from Figure 1A ($r^2$ = 0.46, $P$ = 0.04). The mean lethal aberration frequency was larger for the hyperdiploid cells. When the aberration data in Figure 1A was corrected for chromosome number (corrected frequency = [aberration frequency/average chromosome number] × 46) and expressed as aberrations/diploid chromosome number, the linear relationship between survival and aberration frequency improved ($r^2$ = 0.86, $P = 1.85 \times 10^{-8}$), and the mean lethal aberration frequency now ranged from 0.8 aberrations/cell to 1.4 aberrations/cell with a mean of 1.2 ± 0.1 aberration/cell (Figure 1B). The extrapolation number was 1.1 ± 0.1.

Chromosome number is only a rough approximation of DNA content. Individual chromosomes vary in size and, in tumor cells, are usually highly rearranged. We therefore examined relative DNA content in each cell line. DNA content was determined by flow cytometric analysis of the $G_1$ peak. It is expressed as the $G_1$ DNA content normalized to a diploid lymphoblastoid cell line. There is a direct relationship ($r^2$ = 0.93, $P$ = 0.002) between relative DNA content and chromosome number. As was seen for chromosome number, there is no relationship between DNA content and $SF_2$ ($r^2$ = 0.43, $P$ = 0.16). Using DNA content to correct aberration frequency (corrected frequency = aberration frequency/relative DNA content) results in a mean lethal aberration frequency of 0.9 ± 0.1 aberration/cell.

### DISCUSSION

Taken together, these studies on the relationship between survival and chromosome aberration frequency confirm that aberrations are the primary lethal lesion induced by ionizing radiation, and that the loss of genetic material, presumably through the failure of acentric fragments to segregate properly at anaphase, is the lethal event. While the mean lethal aberration frequency was close to 1 in diploid cell lines, it increased linearly as ploidy increased to approximately 2 in the tetraploid cell lines. Therefore, in hyperdiploid tumour cells, where there is likely to be extensive gene duplication, some genetic loss can be tolerated, and higher levels of chromosome aberrations per cell are required to kill the cell.

In our study, we focused on aberrations induced in $G_1$ cells to make the analysis simpler. Chromosome-type aberrations predominate in $G_1$-exposed cells, while chromatid-type aberrations predominate in cells exposed in $G_2$ or S-phase. Chromatid-type aberrations would be expected to be lethal in only half of the daughter cells. Chromosome aberration frequency was compared to survival measured after the same exposure level in asynchronous populations of cells where approximately half of the exposed cells are in $G_1$ (Schwartz, 1992). Based on the analysis of Quiet et al (1991), we would expect survival levels in $G_1$-exposed cells to be about 20% less than that seen for asynchronous populations. Reducing survival levels by 20% to account for possible radiosensitivity differences between $G_1$-exposed cells and asynchronous populations did not significantly affect our results or conclusions. The mean lethal aberration frequency increased slightly while the extrapolation number decreased slightly.

The failure to find a consistent relationship between DNA ploidy and radiation sensitivity or tumour response to therapy (West, 1994) suggests that there are other factors in addition to ploidy that define response. We and other investigators have identified DNA repair alterations as being an important factor defining radiation sensitivity in tumour cells (reviewed in West, 1994; Schwartz, 1998). In fact, the primary factor underlying the radiosensitivity differences in the eight cell lines studied is alterations in DNA double-strand break rejoining kinetics (Schwartz, 1998). DNA repair alterations, however, define the probability of producing a chromosome aberration. Ploidy defines how many aberrations are required to kill the cell.

As mentioned above, the analysis of chromosome aberration frequency provides a marker of sensitivity that is independent of many other factors including repair. Brown and co-workers (Brown et al, 1992) have proposed that the analysis of chromosome aberration frequency would be a useful parameter to predict radiosensitivity. Our analysis suggests that using chromosome aberration frequency alone without correcting for ploidy could lead to erroneous conclusions. For example, when uncorrected aberration frequency is used to rank cell lines according to their radiosensitivity (Table 2), JSQ-3, SQ-38 and SCC-25 appear more sensitive than clonogenic survival measurements would suggest. Correcting aberration frequency for ploidy yields the correct radiosensitivity ranking. Therefore, the measurement of ploidy should be considered in any battery of predictive assays, especially when the end points do not include clonogenic survival measurements.

 © Cancer Research Campaign 1999

**Table 2** Relative sensitivity as determined by induced chromosome aberration frequency and clonogenic survival[a]

| | Aberrations/cell | Aberrations/diploid chromosome content | $SF_2Gy$ |
|---|---|---|---|
| A549 | 1.0 | 1.0 | 1.0 |
| SQ-20B | 2.8 | 2.8 | 1.6 |
| SCC-12B.2 | 2.9 | 2.6 | 1.3 |
| JSQ-3 | 3.1 | 2.3 | 1.2 |
| SQ-9G | 4.1 | 5.0 | 2.3 |
| SQ-38 | 4.8 | 3.5 | 1.9 |
| SCC-61 | 5.1 | 7.3 | 3.0 |
| SCC-25 | 5.8 | 5.5 | 2.3 |

[a]Relative sensitivity was defined by either 3-Gy-induced chromosome aberration frequency/cell, aberrations/diploid chromosome content, and $SF_2$ Gy. All values are relative to A549 cells.

### ACKNOWLEDGEMENTS

This work was supported by grants GM-51827 (JLS), CA-69044 (JM) and CA-42596 (RRW) from the National Institutes of Health. The authors would like to acknowledge the technical support of Robert Jordan, Linda Wiens, and Michael Beckett, and the helpful comments of Mike Cornforth.

### REFERENCES

Bedford JS (1991) Sublethal damage, potentially lethal damage and chromosomal aberrations in mammalian cells exposed to ionizing radiations. *Int J Radiat Oncol Biol Phys* **21**: 1457–1469

Bedford JS, Mitchell JB, Griggs HG and Bender MA (1978) Radiation-induced cellular reproductive death and chromosome aberrations. *Radiat Res* **76**: 573–586

Brown JM, Evans J and Kovacs MS (1992) The prediction of human tumor radiosensitivity in situ: an approach using chromosome aberrations detected by fluorescence in situ hybridization. *Int J Radiat Oncol Biol Phys* **24**: 279–286

Carrano AV (1973) Chromosome aberrations and radiation-induced cell death. I. Transmission and survival parameters of aberrations. *Mutat Res* **17**: 341–353

Cornforth MN and Bedford JS (1987) A quantitative comparison of potentially lethal damage repair and the rejoining of interphase chromosome breaks in low passage normal human fibroblasts. *Radiat Res* **111**: 385–405

Dewey WC, Miller HH and Leeper DB (1971) Chromosomal aberrations and mortality of x-irradiated mammalian cells: emphasis on repair. *Proc Natl Acad Sci USA* **68**: 667–671

Grote SJ, Joshi GP, Revell SH and Shaw CA (1981) Observations of radiation-induced chromosome fragment loss in live mammalian cells in culture, and its effect on colony forming ability. *Int J Radiat Biol* **39**: 395–408

Hall EJ (1994) *Radiobiology for the Radiologist 4th Edn*. JB Lippincott: Philadelphia

Quiet C, Weichselbaum RR and Grdina DJ (1991) Variation in radiation sensitivity during the cell cycle of two squamous cell carcinomas. *Int J Radiat Oncol Biol Phys* **20**: 733–738

Russell KJ, Wiens LW, Demers GW, Galloway DA, Plon SE and Groudine M (1995) Abrogation of the $G_2$ checkpoint results in differential radiosensitization of $G_1$ checkpoint-deficient and $G_1$ checkpoint-competent cells. *Cancer Res* **55**: 1639–1642

Savage JRK (1975) Classification and relationships of induced chromosomal structural changes. *J Medical Genet* **12**: 103–122

Schwartz JL (1992) The radiosensitivity of the chromosomes of the cells of human squamous cell carcinoma cells. *Radiat Res* **129**: 96–101

Schwartz JL (1998) Chromosome structure alterations and variations in the inherent radiation sensitivity of human cells. *Radiat Res* **149**: 319–324

Szumiel I (1981) Intrinsic radiosensitivity of proliferating mammalian cells. *Adv Radiat Biol* **9**: 281–322

Weichselbaum RR, Rotmensch J, Ahmed-Swan S and Beckett MA (1989) Radiobiological characterization of 53 human tumor cell lines. *Int J Radiat Biol* **56**: 553–560

West CML (1994) Predictive assays in radiation therapy. *Adv Radiat Biol* **18**: 149–180

*© Cancer Research Campaign 1999*
